# Psychometric evaluation of Persian version of medical artificial intelligence readiness scale for medical students

**DOI:** 10.1186/s12909-023-04516-6

**Published:** 2023-07-24

**Authors:** Hossein Rezazadeh, Habibeh Ahmadipour, Mahla Salajegheh

**Affiliations:** 1 Student Committee of Medical Education Development, Education Development Center, Kerman University of Medical Sciences, Kerman, Iran; 2 Community Medicine Department, School of Medicine, Medical Education Leadership and Management Research Center, Kerman University of Medical Sciences, Kerman, Iran; 3 Department of Medical Education, Medical Education Development Center, Kerman University of Medical Sciences, Kerman, Iran

**Keywords:** Reliability, Validity, Questionnaire, Artificial Intelligence, Medical Education

## Abstract

**Background:**

Artificial intelligence’s advancement in medicine and its worldwide implementation will be one of the main elements of medical education in the coming years. This study aimed to translate and psychometric evaluation of the Persian version of the medical artificial intelligence readiness scale for medical students.

**Methods:**

The questionnaire was translated according to a backward-forward translation procedure. Reliability was assessed by calculating Cronbach’s alpha coefficient. Confirmatory Factor Analysis was conducted on 302 medical students. Content validity was evaluated using the Content Validity Index and Content Validity Ratio.

**Results:**

The Cronbach’s alpha coefficient for the whole scale was found to be 0.94. The Content Validity Index was 0.92 and the Content Validity Ratio was 0.75. Confirmatory factor analysis revealed a fair fit for four factors: cognition, ability, vision, and ethics.

**Conclusion:**

The Persian version of the medical artificial intelligence readiness scale for medical students consisting of four factors including cognition, ability, vision, and ethics appears to be an almost valid and reliable instrument for the evaluation of medical artificial intelligence readiness.

## Introduction

The application of artificial intelligence (AI) in medicine has seen significant growth in recent years and holds great potential for improving healthcare outcomes [1]. Studies on AI in the field of medicine and health sciences stated that AI could be of great help to physicians in a more confident diagnosis, improvement of treatment outcomes, and mitigation of medical malpractice [2–6]. For example, artificial intelligence facilitates the diagnosis of coronary heart diseases by echocardiogram analysis [7], the diagnosis of psychotic events and neurological diseases, such as Parkinson’s from speech patterns [8], as well as the diagnosis of polyps and neoplasms in the gastrointestinal system [9]. It even performs certain clinical procedural tasks such as knot tying during robotic surgery [10]. In the near future, artificial intelligence will play a more prominent role in caring for patients and providing them with medical services [3].

Considering the global expansion of artificial intelligence in medicine, it is expected to be one of the main components of medical education in the forthcoming years [1]. Sit et al. (2020) stated that medical students who were trained for artificial intelligence felt more secure in working with artificial intelligence in the future than students who were not [1]. Lindqwister et al. (2020) pointed out that prior knowledge and AI readiness become critical for physicians to perform clinical reasoning better [11]. Liu et al. (2022) found that formal training and current resources for AI are limited in most US medical schools, and there is a definite knowledge gap in AI education in contemporary medical education in the US [12].

Therefore, teaching the principles of artificial intelligence and its application to medical students -as future physicians who would benefit from this technology in their daily clinical practice- becomes very important [13].

Cognitive Constructivism emphasizes on the concept that learning must occur according to a student’s stage of cognitive development [14]. So before starting education, it is important to know the entering behavior of students [15]. Entering behavior describes the prerequisite knowledge, attitudes, or skills which the student already possesses that are relevant to the learning. It refers to what the students have previously learned, their ability, development, and motivational state [16]. Based on pre-assessment, which is one of the basic concepts in education, students’ entering behavior should be measured before the provision of new education [17]. It is of utmost importance since the curriculum planners and faculty plan the lesson content based on learners’ needs after realizing students’ readiness levels in the basic concepts of that subject [18]. In the field of education, preparation is considered an essential part of the teaching and learning process [19]. The emergence of a new behavior change in education depends on students’ level of preparation, so, measuring the level of readiness makes it possible to provide education according to students’ needs from the outset [20]. Therefore, faculty and curriculum developers have to acknowledge the student’s previous knowledge and background about artificial intelligence in medicine which affects their learning.

Park et al. (2021) investigated the United States of America medical students’ perceptions of the impact of artificial intelligence on the practice of medicine. They reported that more than 75% of students pointed to the important role of artificial intelligence in the future of medicine and emphasized the need for formal teaching of artificial intelligence [21]. Yüzbaşıoğlu (2021) investigated the attitudes and perceptions of dental students towards artificial intelligence and its possible applications in this field and demonstrated students’ willingness to improve their knowledge in the AI field [22]. Gray et al. (2022) identified notable gaps in curriculum and educational resources for the use of artificial intelligence in medicine and indicated obstacles, such as a lack of governance structures and processes, resource constraints, and cultural adjustment. These researchers recommended that more studies should be conducted around the world regarding teaching physicians about artificial intelligence [23].

Considering these issues and the importance of investigating medical students’ readiness for the use of artificial intelligence in medicine, it is necessary to study this readiness to plan the next steps of artificial intelligence education in medical sciences universities In Iran. Karaca et al. (2021) designed and psychometrically evaluated a valid and reliable tool for the measurement of medical students’ readiness for the use of artificial intelligence in medicine. Medical Artificial Intelligence Readiness Scale For Medical Students (MAIRS-MS) questionnaire consists of 22 items in four categories including cognition with 8 items, ability with 8 items, vision with 3 items, and ethics with 3 items [24].

Educational practices are dependent on aspects of each context; therefore, the setting is a main factor that can affect psychometric properties in instruments [25]. Consequently, psychometric evaluation of every questionnaire across diverse contexts will strengthen its application as well as create generalizability of the results. Studying medicine in Iran is a doctorate-level degree that requires about seven years of study, research, and limited hands-on practice under the supervision of reputed professors. By holding the M.D. degree, the graduate will be a Doctor in Medicine and can pursue studies in different Specialty programs, which include five years of university studies. Moreover, they can start their professional practice in hospitals, private practices, and clinics [26]. With the current international movement toward artificial intelligence improvement, and Iranian medical universities being no exception, the ability to evaluate the readiness of medical students to use medical artificial intelligence is becoming a crucial component of medical education enhancement. The results of this evaluation are important because they guide various educational design and developmental processes such as curriculum development, instructional design or needs analysis, etc. [24].

The review of the literature demonstrated that so far, the MAIRS-MS questionnaire has not been translated and psychometrically evaluated in the Persian language. This study aimed to translate and psychometric evaluation of the Persian version of the medical artificial intelligence readiness scale for medical students.

## Methods

### Study design and setting

The research was a cross-sectional study that was conducted at the Kerman University of Medical Sciences in Iran between November 2022 and January 2023.

### Participants

The data were collected from 302 undergraduate medical students at Kerman University of Medical Sciences who entered the study by census method. The inclusion criteria were being a medical student in the study period. The exclusion criterion was questionnaires with more than 10% of questions without answers.

### Ethical consideration

The KMU’s institutional review board approved the study (No. IR.KMU.AH.REC.1401.253). The participants did not receive any incentives, and participation was voluntary. Verbal and written consent for participation was obtained based on the proposal approved by the ethics committee. The participants were also assured of the confidentiality of their information, and it was explained that the results would only be used for research objectives.

### Translation process

The translation process was performed according to the backward-forward translation procedure [27]. Firstly, two English language experts separately translated the questionnaire into Persian. One of them had expertise in translating medical texts and the other had expertise in translating colloquial expressions. They were not aware of the structure of the instrument. Secondly, two medical education faculty members and two artificial intelligence specialists compared both translated versions, and made the necessary revisions in terms of ambiguous words, sentence structure, and meaning of the sentence. Thirdly, two English language experts investigated and confirmed the changes. Fourthly, the translated versions were reviewed and compared with the original instrument by two medical education faculty members and two artificial intelligence specialists in terms of conceptual, semantic, and content equivalence. The instructions for answering the questionnaire were also checked. Finally, the pre-final version was compiled.

### Psychometric evaluation

#### Content validation

The content validity of the initial MAIRS-MS was investigated both quantitatively and qualitatively by expert opinion. Ten experts from faculty members were recruited based on their experience in artificial intelligence and their expertise in medical education. They were selected within Kerman University of Medical Sciences. Experts were asked to consider each item of the MAIRS-MS based on the criteria of “essential,” “relevance,” “clarity,” and “simplicity.” Each item was assessed using Likert scales: A three-point scale for “essential” (1 – unessential, 2 – useful, but not essential, and 3 – essential,), and four-point scales for “relevance” (1 – not relevant, 2 – rather relevant, 3 – relevant, and 4 – completely relevant) and “clarity” (1 – not simple, 2 – rather simple, 3 – simple and 4 –completely simple) criteria. In addition, the experts were asked to provide comments about the “simplicity” of each item (fluency and using simple and understandable words) as well as the most appropriate placement and order of the items.

We examined content validity by computing Content Validity Ratio (CVR) and Content Validity Index (CVI) using ratings of item relevancy that were highlighted by the content experts. Given the ten experts who evaluated the items, the minimum acceptable amount of CVR was 0.62 based on the Lawshe table [28]. The formula for calculating CVI in Waltz and Bausell’s method is the number of all the respondents in the “relevancy,” “clarity,” and “simplicity” criteria divided by the number of experts who have scored 3 or 4 in the relevant question in that criterion. In this formula, if an item has a score of more than 0.79 that item is retained in the questionnaire. If CVI is between 0.70 and 0.79, the item is questionable and needs correction and revision. Furthermore, if it is less than 0.70, the item is unacceptable and it must be deleted [29]. The corrective comments of experts about the wording of items, such as fluency, using simple and understandable words, and the suitable placement of the words were used.

#### Face validation

Students’ opinions were used to check the face validity. In this regard, interviews were conducted with ten medical students using concurrent verbal probing and thinking aloud. The questionnaire items were examined in terms of fluency, appropriate phrasing, avoiding specialized words, and potential ambiguity.

#### Construct validation

The modified MAIRS-MS based on content and face validation was sent to 302 medical students who were included in the study by census method. The sample size chose based on the recommendation for confirmatory factor analysis of 5–10 people per parameter estimate in the measurement model [30]. The questionnaire was sent out two additional times with a gap of approximately two weeks between each distribution. The delivery methods included email and follow-ups through social media.

A Confirmatory Factor Analysis (CFA) was performed to examine and verify the assumed four factors structure of the MAIRS-MS with LISREL software (8.8 version. New Jersey). Several fit indices were carried out to assess the fit of the hypothesized model to the data. Relative/normed chi-square (χ2/df) range from as high as 5.0 to as low as 2.0, Root mean square error of approximation (RMSEA) below 0.08, standardized root mean square residual (SRMR) less than 0.08, Comparative fit index (CFI), Normed-fit index (NFI), and Non-Normed Fit Index (NNFI) greater than 0.95 were recommended. Since it is not requisite or reasonable to include all indices and according to Hu and Bentler’s Two-Index Presentation Strategy, a combination rule of CFI of 0.96 or higher and a SRMR of 0.09 or lower was considered herein [31]. Also, the convergent validity was assessed by the calculation of Average Variance Extracted (AVE), and Composite Reliability (CR).

#### Reliability assessment

The internal consistency of the MAIRS-MS was investigated by Cronbach’s alpha. Internal consistency of more than 0.7 was considered suitable [32].

## Results

### Demographic data

All 10 experts completed the content validation form. Most of them were women (75%). Half were affiliated to medical education and most were assistant professors. 302 medical students participated in investigating construct validity most of them (70%) were interns, 23% were clerkships and the rest were in the basic sciences stage.

### Content validity

The overall CVR was 0.75, which was acceptable. The CVI for all items was 0.92 by using the Waltz and Bausell method (In terms of relevance 0.96, clarity 0.90, and simplicity 0.90). Two items (I can explain the AI applications used in healthcare services to the patient) and (I can explain the limitations of AI technology) with CVR ˂0.70 were removed as they were identified as being vague or similar to other items.

### Face validity

Based on the students’ opinions in the face validation process, all translated items were clear and accepted.

### Construct validity

In CFA, the factor loadings of the items were between 0.52 and 0.97, therefore, none of the items were deleted (Table 1). A standardized root means square residual (SRMR) = 0.09 and Comparative Fit Index (CFI) = 0.95, indicating a fair fit of the model based on Hu and Bentler’s Two-Index Presentation Strategy. Other fit indices were as the followings; (χ2 /df = 5.5, RMSEA = 0.1, NFI = 0.94, =, and NNFI = 0.94)

Table 1 shows Cronbach’s alpha coefficients, Average Variance Extracted (AVE), and Composite Reliability (CR) of the four-factor model of the Persian version of the medical artificial intelligence readiness scale for medical students (MAIRS-MS). According to this table, all subscales and also whole questionnaire (α = 0.94) had appropriate internal consistency. Also, the Persian version of MAIRS-MS had suitable convergent validity (AVE > 0.5, CR > 0.7, and AV > CR).

**Table 1:** The factor loading, Cronbach’s alpha coefficients, Average Variance Extracted (AVE), and Composite Reliability (CR) of the four-factor model of the Persian version of the medical artificial intelligence readiness scale for medical students (MAIRS-MS)

Factor	Item	Loading	Cronbach’s alpha coefficients	AVE	CR
Cognition	1	0.80	0.91	0.61	0.92
	2	0.59			
	3	0.85			
	4	0.83			
	5	0.82			
	6	0.89			
	7	0.76			
	8	0.62			
Ability	9	0.87	0.92	0.70	0.95
	10	0.91			
	11	0.89			
	12	0.80			
	13	0.80			
	14	0.52			
	15	0.81			
	16	0.79			
Insight	17	0.88	0.89	0.82	0.93
	18	0.92			
	19	0.79			
Ethics	20	0.78	0.92	0.87	0.95
	21	0.97			
	22	0.90			

### Production of the final questionnaire

After investigating reliability and validity, the Persian version of MAIRS-MS with 20 items in four domains was finalized. These domains included “cognition” with 8 items, “ability” with 7 items, “vision” with 2 items, and “ethics” with 3 items.

## Discussion

Considering the critical importance of learning artificial intelligence for medical students, the increasing advancement of this science in medical sciences, and the lack of a valid and reliable tool in the Persian language to measure medical students’ readiness for the use of artificial intelligence in medicine, the results of the present study indicated that the MAIRS-MS questionnaire can be used as a valid and reliable instrument to measure medical students’ readiness for artificial intelligence training in four domains of cognition, ability, vision, and ethics.

Despite being recently developed, this instrument has been used several times in different contexts. Laupichler et al. (2022) assessed the effect of a flipped classroom course to foster medical students’ AI literacy with a focus on medical imaging and reported that the MAIRS-MS questionnaire is a practical and appropriate tool. The results demonstrated that participation in this course led to an increase in medical students’ readiness for the use of artificial intelligence in medical imaging [33]. Gray et al. (2022) introduced this questionnaire as a valid instrument for measuring medical students’ readiness for teaching AI in medicine [23]. Aboalshamat et al. (2022) assessed levels of readiness for AI among medical and dental students and graduates in Saudi Arabia with the MAIRS-MS. They concluded that participants had low levels of AI readiness. It was recommended that artificial intelligence education should be provided from the early years of education in medicine [34]. Xuan et al. (2022) measured AI readiness among medical students using MAIRS-MS in Malaysia. They recommended that education policymakers should set up more artificial intelligence training courses to provide and introduce basic concepts of artificial intelligence, especially for general medicine students. This enables them to gain more confidence in interacting with artificial intelligence technology in the future [35].

Due to the novelty of artificial intelligence in medicine and its application in the treatment of patients, several studies have been conducted to investigate the various aspects of this technology across the globe. These studies, which are often performed based on a quantitative research approach, have used different and numerous instruments for data collection. Nonetheless, the validity of these tools and their design method are debated issues.

Boillat et al. (2022) investigated the familiarity of physicians and medical students with artificial intelligence in medicine using a researcher-made questionnaire. This questionnaire was designed only by reviewing the literature and did not use experts’ opinions. Moreover, for psychometric analysis, only the reliability of the questionnaire was assessed by calculating Cronbach’s alpha coefficient [36]. Doumat et al. (2022) investigated the knowledge and attitude of medical students regarding artificial intelligence in medicine in Lebanon using a researcher-made questionnaire. This tool included 15 items on knowledge and 5 questions assessing attitude. However, no information was provided regarding the design method, as well as the results of the validity and reliability of this questionnaire [37]. Jha et al. (2022) investigated medical students’ knowledge of artificial intelligence, the role of artificial intelligence in medicine, and the priorities related to its education in Nepal using a questionnaire. The face validity of the tool was assessed on 20 graduates, and its reliability was verified by computing Cronbach’s alpha coefficient which was reported as 0.6 [38].

The reliability of the Persian version of MAIRS-MS was confirmed by calculating Cronbach’s alpha coefficient of 0.94, demonstrating a higher level compared to the reliability of the original version of the original instrument (0.87). Moreover, in the categories of cognition, ability, vision, and ethics, the reliability coefficients were reported as 0.91, 0.92, 0.89, and 0.92, respectively. As illustrated, the reliability of the Persian version was higher compared to the English questionnaire in all four areas. One of the other strengths of this study was confirmatory factor analysis on 302 medical students. This population is much more than the required sample size recommended for factor analysis.

This study has some limitations which must be considered. All participants were from one university in Iran. This sampling bias might undermine the external validity of the results and cause selection bias. Due to the fair fit of the model according to the fit indices and to be used in another context the MAIRS-MS needs further validation in groups speaking other languages, different cultures, and in other universities.

Although this tool was designed for medical students, it can also be used to measure AI readiness in medicine in other populations, such as physicians and residents. Therefore, it is suggested that future studies evaluate different samples of medical science specialists. Furthermore, MAIRS-MS can be of great help to educational planners and policymakers as a valuable tool in the design of artificial intelligence-related curricula tailored to students’ needs. This instrument can also be useful in measuring the effectiveness of training courses related to artificial intelligence.

## Conclusion

Considering the excellent reliability, appropriate convergent validity, and almost acceptable construct validity of the Persian version of the MAIRS-MS, as well as its conciseness and ease of implementation, it can be used to evaluate medical students’ readiness for the use of artificial intelligence in medicine. The outcomes of this evaluation hold significance as they steer diverse educational creation and growth procedures, like devising curricula, formulating instructional designs, or conducting needs analyses.

## Data Availability

The datasets used and/or analyzed in the current study are available from the corresponding author upon reasonable request.
